# Protein Coding and Long Noncoding RNA (lncRNA) Transcriptional Landscape in SARS-CoV-2 Infected Bronchial Epithelial Cells Highlight a Role for Interferon and Inflammatory Response

**DOI:** 10.3390/genes11070760

**Published:** 2020-07-07

**Authors:** Radhakrishnan Vishnubalaji, Hibah Shaath, Nehad M. Alajez

**Affiliations:** 1 Cancer Research Center, Qatar Biomedical Research Institute (QBRI), Hamad Bin Khalifa University (HBKU), Qatar Foundation (QF), Doha 34110, Qatar; vbradhakrishnan@hbku.edu.qa (R.V.); hshaath@hbku.edu.qa (H.S.); 2 College of Health & Life Sciences, Hamad Bin Khalifa University (HBKU), Qatar Foundation (QF), Doha 34110, Qatar

**Keywords:** SARS-CoV-2, COVID-19, gene expressions, pathway analysis, bronchial epithelial, IFN response, immune response, MAPK, lncRNAs

## Abstract

The global spread of COVID-19, caused by pathogenic severe acute respiratory syndrome coronavirus 2 (SARS-CoV-2) underscores the need for an imminent response from medical research communities to better understand this rapidly spreading infection. Employing multiple bioinformatics and computational pipelines on transcriptome data from primary normal human bronchial epithelial cells (NHBE) during SARS-CoV-2 infection revealed activation of several mechanistic networks, including those involved in immunoglobulin G (IgG) and interferon lambda (IFNL) in host cells. Induction of acute inflammatory response and activation of tumor necrosis factor (TNF) was prominent in SARS-CoV-2 infected NHBE cells. Additionally, disease and functional analysis employing ingenuity pathway analysis (IPA) revealed activation of functional categories related to cell death, while those associated with viral infection and replication were suppressed. Several interferon (IFN) responsive gene targets (IRF9, IFIT1, IFIT2, IFIT3, IFITM1, MX1, OAS2, OAS3, IFI44 and IFI44L) were highly upregulated in SARS-CoV-2 infected NBHE cell, implying activation of antiviral IFN innate response. Gene ontology and functional annotation of differently expressed genes in patient lung tissues with COVID-19 revealed activation of antiviral response as the hallmark. Mechanistic network analysis in IPA identified 14 common activated, and 9 common suppressed networks in patient tissue, as well as in the NHBE cell model, suggesting a plausible role for these upstream regulator networks in the pathogenesis of COVID-19. Our data revealed expression of several viral proteins in vitro and in patient-derived tissue, while several host-derived long noncoding RNAs (lncRNAs) were identified. Our data highlights activation of IFN response as the main hallmark associated with SARS-CoV-2 infection in vitro and in human, and identified several differentially expressed lncRNAs during the course of infection, which could serve as disease biomarkers, while their precise role in the host response to SARS-CoV-2 remains to be investigated.

## 1. Introduction

Coronavirus disease 2019 (COVID-19), caused by severe acute respiratory syndrome coronavirus 2 (SARS-CoV-2) was declared a global pandemic by the World Health Organization (WHO) on 11 March 2020 [1,2]. Since its initial emergence, COVID-19 has spread to the majority of countries worldwide, with more than 10.0 million confirmed cases and over 500,000 deaths thus far [3]; requiring an imminent response from medical research communities to better understand this rapidly spreading infection.

Although SARS-CoV-2 is the seventh coronavirus which has led to human disease, it is the third strain to surpass common cold like symptoms, all three deriving from the β-coronavirus genus [4], with the first and second resulting in endemics in recent history [5]. Severe acute respiratory syndrome coronavirus (SARS-CoV) and the Middle East respiratory syndrome coronavirus (MERS-CoV) breakouts were reported in Southern China in 2003 and Saudi Arabia in 2012, respectively. To confirm genealogy, genomic sequencing of SARS-CoV and MERS-CoV showed approximately 79% and 50% similarities with SARS-CoV-2, respectively [6]. SARS-CoV and SARSCoV-2 also share around 76% in amino acid identities [7]. Mathematical algorithms show that SARS-CoV-2 has a high estimated basic reproductive number (R_0_), reported as high as 6.49 (mean; 3.28), exceeding WHO estimations of 2.5 [8]. In contrast, SARS-CoV and MERS-CoV have R_0_ scores of <1 and 1.4–2.5, respectively [6]. This is indicative of SARS-CoV-2 ability to spread faster than other *Coronaviridae* strands, possibly as a result of asymptomatic transmission when encountering close human contact (respiratory droplets) during the incubation period of 2 to 14 days [6]. Symptoms of COVID-19 include atypical pneumonia, high fever, respiratory difficulties, shortness of breath, a dry cough and a sore throat. Severe cases have also reported organ failure and death. Most of these same symptoms also present with other viruses of the same *Coronaviridae* family.

Structurally, coronaviruses are single stranded RNA, positive sense, enveloped viruses of around 30 kilobases, coding for multiple structural and non-structural proteins [9]. The four structural proteins include the main lattice forming membrane (M) protein, spike proteins (S) which form dimers and facilitate viral attachment onto host cell receptors via glycoprotein projections, envelop (E) proteins and the nucleocapsid (N) protein which homes the helical viral genome, some of which is non-coding [10]. With viral infections initiating from the binding to host cellular receptors, extensive research has been conducted on receptor recognition, identifying human angiotensin converting enzyme 2 (hACE2) as receptor for human viral entry for SARS-CoV and human dipeptidyl peptidase 4 (hDPP4) as receptor for MERS-CoV [11,12,13,14]. With this knowledge at hand, researchers were able to swiftly associate SARS-CoV-2 binding with hACE2, mainly expressed on type 2 alveolar cells [15], as the mechanism of entry into host cells due to amino acid residue similarities in its receptor binding domain (RBD). In fact, nine out of the 14 residues in the RBD of SARS-CoV are conserved in human through bat [16]. Residues evolving on the viral RBD is predicted to contribute to viral adaptation, in turn, facilitating cross species transmission [17,18,19]. Comparative sequence analysis in human ACE2 revealed 84.8% and 80.8–81.4% similarities with pangolins and bats, respectively [20]. Further interspecies analysis reports are constantly emerging [21,22] to solidify our understanding of the origins of SARS-CoV-2, facilitating helpful solutions for subsequent disease control. In fact, through structural and biochemical data, Shang et al., have shown that through these changes in amino acid residues, SARS-CoV-2 RBD has a significantly higher hACE2-binding affinity than SARS-CoV RBD [23,24]. This could be due to the identification of naturally selected “hotspot” regions of the RBD for exceptionally high affinity hACE2 binding [19]. Cryogenic electron microscopy (Cryo-EM) structures of the SARS-CoV-2 spike glycoprotein protrusions show that 20 out of 22 are conserved from SARS-CoV to SARS-CoV-2, with an additional furin cleavage site setting them apart.

Aside from the coding part of the human genome, research efforts in the past decade have been significantly shaped by the discovery of non-coding RNAs’ (ncRNAs) potential and role in various cellular processes, including the onset and progression of many diseases including cancers and infectious disease [25,26]. Viral ncRNAs have shown to have just as much functional and pathological importance as viral structural proteins [27,28]. Deep RNA sequencing of lung tissue of SARS-CoV infected mice revealed three small viral RNAs (svRNAs) that when inhibited via the use of antagomirs, caused a reduction in pro-inflammatory cytokine expression and reduced lung pathology [29]. Phenomenal changes in ncRNA expression are also seen within host cells, which can play a major role in respiratory virus pathogenesis, with long non-coding RNAs (lncRNAs) exhibiting higher tissue specificity than coding genes [30]. LncRNAs are around 200 base pairs in length and have been predicted to play a role in innate immune responses via their association with IFN mechanistic pathways [31]. Understanding the effects of their differential expression and modes of action will immensely impact the fields of immunology and infectious diseases. Whole transcriptome analysis of host response to SARS-CoV in mouse strains highlighted over 500 differentially expressed annotated lncRNAs, which clearly showed association with innate immune signaling and pathogenesis regulation through Signal transducer and activator of transcription 1 (STAT1) [31].

In our current study, we employed modern computational genomics tools and performed more in-depth analysis of transcriptome data from primary normal human bronchial epithelial cells (NHBE) during the course of SARS-CoV-2 infection and lung biopsies derived from COVID-19 patients from the Blanco-Melo et al. study, revealing multiple affected mechanistic networks and functional categories related to innate immunity, interferon activation and cellular response to viral infection [32]. Additionally, we characterized the lncRNA transcriptional portrait in response to SARS-CoV-2 infection in NHBE cells, as well as in lung biopsies derived from COVID-19 patients. To our knowledge, this is the first study highlighting systemic alterations in cellular host response to SARS-CoV-2 infection in the context of lncRNAs, adding to previously published data, in efforts to further grasp their potential utilization as disease markers or therapeutic targets.

## 2. Materials and Methods

### 2.1. Dataset and Bioinformatics

Raw RNA sequencing data were retrieved from the sequence read archive (SRA) database under accession no. (PRJNA615032) [32]. Detailed experimental procedures are explained in the aforementioned reference. Single-end FASTQ files for mock (SRX8089292, SRX8089293, and SRX8089294) and SARS-CoV-2 infected (SRX7990869, SRX7990870, and SRX7990871) NHBE cells (24 h) as well as FASTQ files for lung biopsies from healthy (SRX8089341 and SRX8089342) and from patients with COVID-19 (SRX8089343 and SRX8089344) were retrieved using the SRA toolkit version 2.9.2 as previously described [33]. FASTQ files were subsequently mapped and aligned to the hg38 reference genome (including both protein coding and non-coding RNAs) using built in RNA-seq analysis module in CLC genomics workbench 20.0 with default settings as we described before [34]. To detect viral genome transcriptome, raw sequence data were aligned to the NCBI viral genome reference sequence in CLC genomics workbench 20.0 and subsequently mapped reads to severe acute respiratory syndrome coronavirus 2 isolate Wuhan-Hu-1, complete genome (NCBI Reference Sequence: NC_045512.2) was estimated. All expression values for mRNA, lncRNA, and viral genes from the current study are provided in Supplementary Table S1. Normalized expression data (TPM (Transcripts Per Million) mapped reads) for protein coding were separated from those of lncRNAs using BioMart Ensembl tool and were sequentially imported into AltAnalyze v.2.1.3 software for differential expression analysis using 2.0-fold change and <0.05 *p*-value cut-off. Transcripts with raw expression values < 1.0 TPM were excluded from the analysis. Hierarchical clustering was performed using cosine for columns and cosine for rows, and marker finder prediction as described before [35,36]. Cell type and gene expression explanations were allotted using a custom gene-set enrichment approach. Cell type predictions were created from the software Gene Ontology (GO)-Elite in AltAnalyze using its previously defined cell and tissue marker gene database. This database encompasses markers for lots of nominated cell types and bulk tissue samples. The databases are created mainly from the Ensembl database that includes all external ID systems related to Ensembl as well as supported platforms (e.g., Illumina). Relationships to numerous biological Ontology (GO, disease and phenotype), pathway and gene set resources are conserved. Marker Finder analysis was achieved within each experimental condition to predict specific markers based on gene-set enrichment by GO-Elite algorithm as described before [37].

### 2.2. Gene Set Enrichment and Modeling of Gene Interactions Networks

Upregulated genes were imported into the Ingenuity Pathways Analysis (IPA) software (Ingenuity Systems; Qiagen, Redwood City, CA, USA) www.ingenuity.com/) and were subjected to functional annotations and regulatory network analysis using upstream regulator analysis (URA) to analyze upstream molecules, which are connected to genes in the dataset via a set of either direct or indirect relationship based on changes in expression. Mechanistic networks (MN) analysis was performed using IPA to generate signaling cascades which connect upstream regulators to help visualize how they connect to explain the observed changes in gene expression. Downstream effector analysis (DEA) identifies the biological processes (disease) and functions, which are casually affected by deregulation of genes in datasets and predicts their activation state (Z score). IPA uses precise algorithms to predict functional regulatory networks from gene expression data and provides a significance score for each network according to the fit of the network to the set of focus genes in the database. The score represents the negative log of the *p*-value for the probability that focus genes in the network are found together by chance [34].

### 2.3. Statistical Analyses

Statistical analyses and graphing were performed using Microsoft excel 2016 and GraphPad Prism 8.0 software (GraphPad, San Diego, CA, USA). Two tailed *t*-test was used for comparative groups. *p*-values ≤ 0.05 (two-tailed *t*-test) were considered significant. For IPA analyses, a Z score (−2.0 ≥ Z ≥ 2.0) was considered significant.

## 3. Results

### 3.1. Identification of Differentially Regulated Host Cell Genes in Response to SARS-CoV-2 Infection NBHE Cells

**Figure 1:**
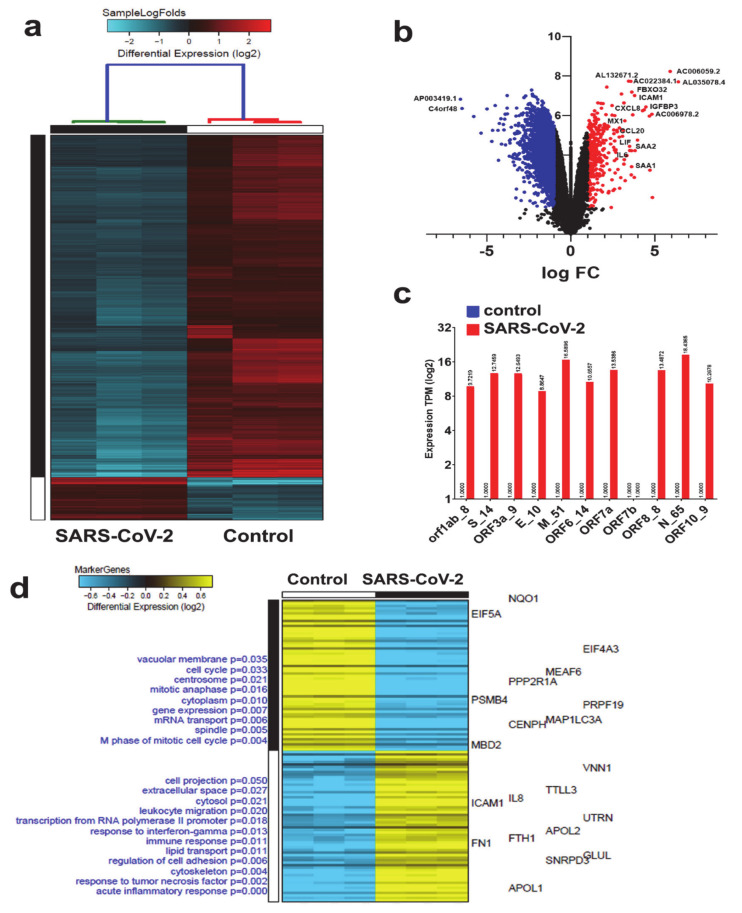
Clustering of SARS-CoV-2 and control NBHE cells based on mRNA RNA-seq analysis. (**a**) Hierarchical clustering of control and SARS-CoV-2 infected NHBE cells based on differentially expressed mRNAs. Each column represents one replica, while each row represents one mRNA. Expression is depicted at the indicated color scales. (**b**) Volcano plot representation of significantly altered genes in NHBE SARS-CoV-2 vs. mock infected cells. Red and blue colors indicate the genes with significantly increased or decreased expression, respectively. (**c**) Relative expression of the indicated viral genes in SARS-COV-2 NHBE vs. control NHBE cells. (**d**) Marker discovery analysis to identify putative markers that are selectively expressed in control vs. SARS-CoV-2 infected NBHE cells. Enriched gene ontology (GO) associations are indicated on the *y* axis.

To highlight the changes in cellular host genes in response SARS-CoV-2 infection, we employed recent RNA-seq datasets utilizing the NBHE cell model. Analysis of three biological triplicates of NHBE cells mock treated (control) or infected with SARS-CoV-2 (USA-WA1/2020) instigated differential expression of 377 upregulated and 3012 downregulated mRNAs (Figure 1a and Supplementary Table S2). A volcano plot (scatterplot) that shows statistical significance (log *p* value; *Y*-axis) vs. magnitude of change (log fold change; *X*-axis) is depicted in Figure 1b, with selected genes being displayed. The red colors (right) and blue colors (left) represent genes with significantly upregulated or downregulated expression in NHBE SARS-CoV-2 vs. mock-infected cells, respectively (Figure 1b). Transcriptome data were subsequently mapped and aligned to the NCBI viral genome reference sequence which revealed the expression of several SARS-CoV-2 viral genes, particularly Open Reading Frame (ORF) that codes for viral non-structural proteins (NSP), orf1ab_8; S_14; ORF3a_9; E_10; M_51; ORF6_14; ORF7a; ORF8_8; N_65; and ORF10_9, except ORF7b which was not expressed (Figure 1c).

We subsequently employed the marker gene finder algorithm to identify genes associated with the SARS-COV-2, employing the NHBE model. Modules of co-expressed mRNAs (120 genes) were associated with specific biological condition, (i.e., SARS-CoV-2), where the heatmap highlights differentially expressed mRNAs that are positively correlated with an idealized cluster-specific expression profile. We found putative markers that are selectively expressed in SARS-CoV-2 vs. control infected NBHE cells. The text on the left side of the *y*-axis indicates enriched cell- type markers from the default gene-set enrichment analysis (Gene Ontology-Elite) along with top default gene markers for each GO term displayed on the right side of the *y*-axis on the heatmap. The color scale displays differential gene expression (log2). In SARS-COV-2 infected cells, top modules of co-expressed genes were associated with acute inflammatory response, response to TNF and Interferon gamma (INFG), immune response, cell adhesion, leukocyte migration, cell projection, lipid transport, transcription from RNA polymerase II promotor, cytosol and extra cellar space, and cell projection, respectively. Whereas cell cycle, mitosis, vascular membrane and mRNA transport, and cellular development GO classifications were enriched in the mock control group (Figure 1d and Supplementary Table S3).

### 3.2. Canonical and Upstream Regulator Analysis Highlights Activation of Interferon Response Pathways in SARS-CoV-2 NHBE Cells

**Figure 2:**
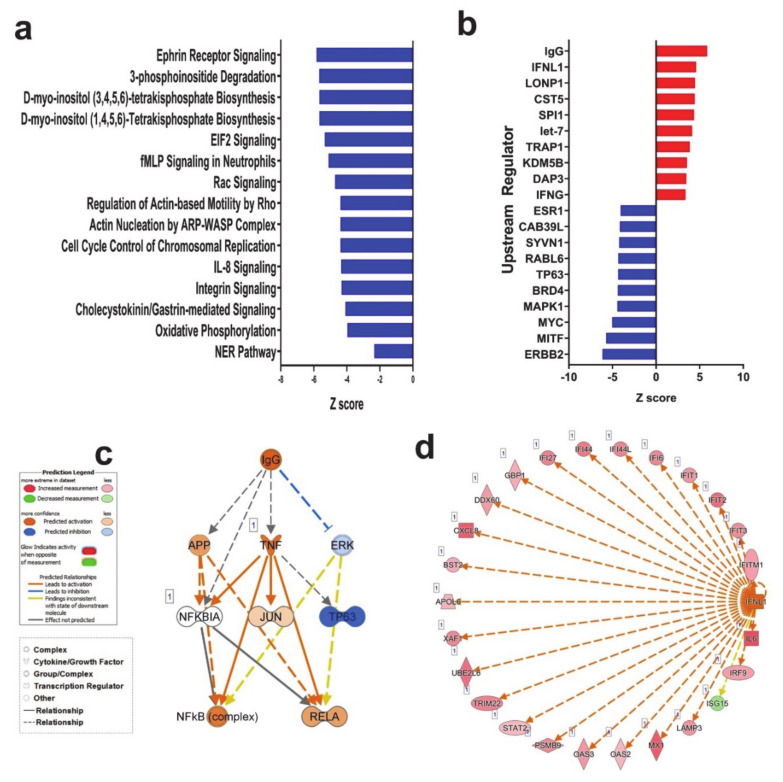
Ingenuity pathway analysis (IPA) of differentially expressed gene in Control and SARS-CoV-2 infected NBHE cells. (**a**) Canonical IPA analysis of differentially expressed genes in SARS-CoV-2 vs. control NBHE cells. *X*- axis indicates Z score while *y*-axis indicates the corresponding canonical pathways. Blue color indicates suppressed pathways. (**b**) Upstream regulator analysis of differentially expressed genes in SARS-CoV-2 vs. control NBHE cells using IPA. IgG (**c**) and interferon lambda 1 (IFNL1, (**d**)) mechanistic network and their activation state in SARS-CoV-2 infected NBHE cells based on IPA analysis.

Canonical pathway analysis on the differentially expressed genes in SARS-CoV-2 NHBE cells using ingenuity pathway analysis (IPA) revealed downregulation of several canonical pathways, including Ephrin receptor signaling (Figure 2a and Supplementary Table S4). IPA upstream regulator analysis provides a powerful tool to predict the deregulated functional activities that are possibly affected by the transcriptome data. Figure 2b horizontal bars denote the different upstream regulators (top 10 up and down regulated genes) in the SARS-CoV-2 group based on the Z-scores. This analysis revealed significant enrichment in several functional classifications, those predicted to be activated (top 10) are associated with type I and III interferon groups that play important roles against viral infection, mainly IFNL1 (IL-29; IFN-lambda 1), IFNG cytokines and IgG complex, and LONP1, CST5, SPI1, TRAP1, KDM5B, DAP3, and let-7 microRNA (miR/miRNA). Conversely, functional categories that were predicted to be inhibited (top 10) are interrelated to p53 binding and cell cycle (ESR1, CAB39L, SYVN1, RABL6, TP63, BRD4, MAPK1, MYC, MITF and ERBB2 (Figure 2b and Supplementary Table S5).

IgG complex and IFNL were observed among the top predicted upstream regulators in the SARS-CoV-2 group and were chosen for mechanistic network analysis to provide better understanding of the upstream regulators leading to downstream effector molecules and subsequent changes in gene expression. The predicted active state of IgG complex was anticipated to activate the NFkB and RELA complex through inhibiting ERK directly. Although mechanistic network analysis illustrates that the IgG complex plays a role in the downregulation of TP63 and activation of RELA through activation of TNF, the effect of this relationship is still under evaluation (Figure 2c). Likewise, the predicatively active IFNL and its associated network molecules, are illustrated in Figure 2d.

### 3.3. SARS-CoV-2 NHBE Gene Signature Predicted Activation of Cell Death and Inhibition of Viral Infection Based on Disease Functions Analysis by IPA

**Figure 3:**
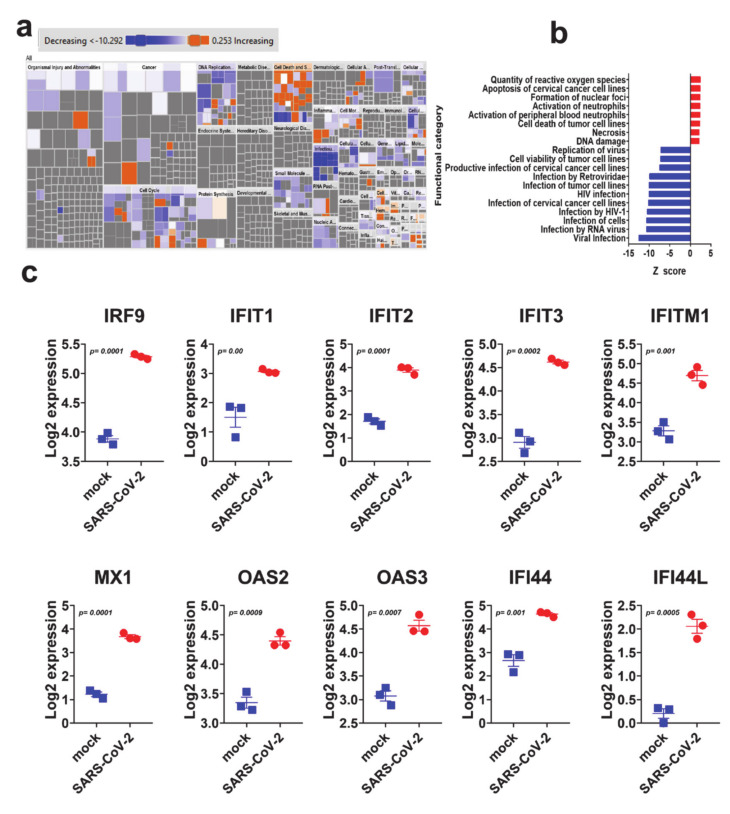
Downstream effector analysis of differentially expressed genes in SARS-CoV-2 infected NBHE cells. (**a**) Tree map (hierarchical heat map) depicting affected functional categories based on differentially expressed genes where the major boxes represent a category of diseases and functions. Each individual colored rectangle is a particular biological function or disease and the color range indicates its predicted activation state—increasing (orange) or decreasing (blue). Darker colors indicate higher absolute Z-scores. In this default view, the size of the rectangles is correlated with increasing overlap significance. (**b**) Bar graph depicting the activated (red) and suppressed (blue) functional categories. (**c**) Expression of selected gene from the antiviral defense genes category in mock and SARS-COV-2 infected NHBE cells.

Downstream effector analysis in IPA predicts the differences in the downstream biological functions that are presumed to be affected by the changes in the transcriptome. Tree map (hierarchical heat map) portrays the affected downstream functional groups based on differentially expressed mRNAs, where the major boxes represent a category of diseases and functions in the SARS-CoV-2 group. Each individual rectangle (blue and orange colors) indicates the decreasing and increasing state, while rectangle dimensions using Fisher exact test (FET) *p*-value is associated with increasing overlap significance, either up or down as a group, and color intensity stipulates higher absolute Z-scores (Figure 3a). Disease and function analysis on the differentially expressed genes revealed the most significant enrichment in pathways related to reactive oxygen species, induction of apoptosis and necrosis, as well as activation of neutrophils in SARS-CoV-2 infected NHBE cells (Figure 3a,b). On the other hand, the most suppressed functional category in SARS-CoV-2 infected NHBE cells was the infectious disease category, which includes replication and infection by viruses (Supplementary Table S6).

IPA analysis revealed the suppression of viral infection and replication as the main affected functional category in SARS-CoV-2 NHBE cells. Therefore, we sought to elucidate the expression of selected genes with known role in combating viral infection. Figure 3e depicts the upregulation of IRF9, IFIT1, IFIT2, IFIT3, IFITM1, MX1, OAS2, OAS3, IFI44 and IFI44L in SARS-CoV-2 infected NBHE cells, which are known to play crucial roles in host cell responses to viral infection through type I interferon. Those data were further validated in RNA-seq dataset from calu-3 (lung adenocarcinoma) cells infected with SARS-CoV-2 (Supplementary Figure S1).

### 3.4. Expression Pattern of lncRNAs in SARS-CoV-2 Infected NBHE Cells

**Figure 4:**
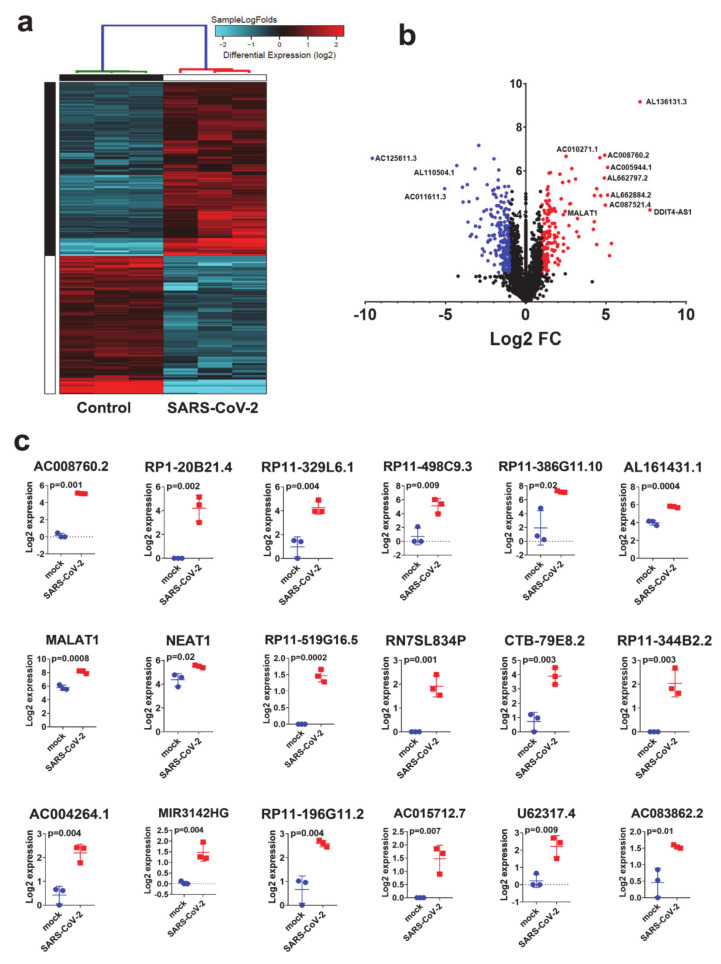
Clustering of SARS-CoV-2 and control NBHE cells based on lncRNA expression. (**a**) Hierarchical clustering of control and SARS-CoV-2 infected NHBE cells based on differentially expressed lncRNAs. Each column represents one replica, while each row represents one lncRNA. Expression is depicted at the indicated color scale. (**b**) Volcano plot representation of differential expression analysis of lncRNAs in NHBE SARS-CoV-2 vs. mock infected cells. Red and blue colors indicate the genes with significantly increased or decreased expression, respectively. (**c**) Expression of selected lncRNAs in mock and SARS-COV-2 infected NHBE cells.

Given their emerging role as key regulators of various biological functions [38], our comparative analysis comparing SARS-CoV-2 and control NBHE cells revealed 155 upregulated and 195 downregulated lncRNAs in response to SARS-CoV-2 viral infection (Supplementary Table S7). While lncRNAs are emerging regulators implicated in a myriad of biological processes; their role in antiviral host response is mostly unknown. Hierarchical clustering of control and SARS-CoV-2 infected NHBE cells based on differentially expressed lncRNAs revealed a clear separation of the two treatment groups (Figure 4a). Volcano plot illustrating differentially expressed lncRNAs in SARS-CoV-2 vs. mock infected NHBE cells is presented in Figure 4b.The expression level of selected upregulated lncRNAs from the RNA-seq data (AC008760.2, RP1-20B21.4, RP11-329L6.1, RP11-498C9.3, RP11-385G11.10, AL161431.1, MALAT1, NEAT1, RP11-519G16.5, RN7SL834P, CTB-79E8.2, RP11-344B2.2, ACOO4264.1, MIR3142HG, RP11-198G11.2, AC015712.7, U62317.4 and AC083862.2) in SARS-CoV-2 infected NBHE cells is presented in figure (Figure 4c). The role of those lncRNAs in the cellular response to SARS-CoV-2 remains to be investigated.

### 3.5. Alterations in Gene Expression and Associated Functional Classification in Lung Tissue from Patient with COVID-19 Revealed Activation of Interferon and Response to Viral Infection as the Main Hallmarks

**Figure 5:**
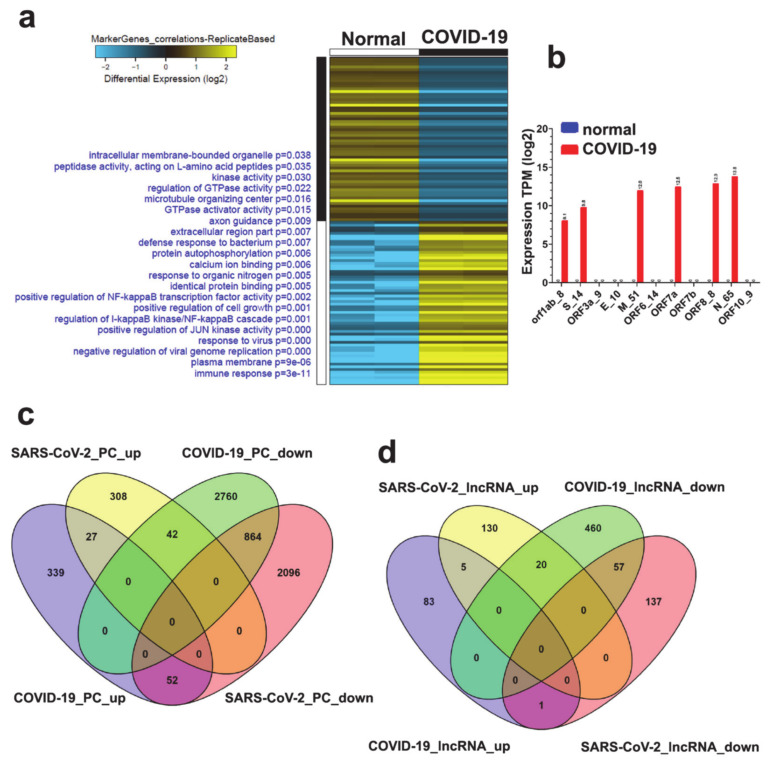
Alteration in mRNA and lncRNA expression in lung biopsies from patient with COVID-19. (**a**) Marker discovery analysis to identify putative markers that are selectively expressed in the lungs from COVID-19 subjects compared to normal lung tissue. Enriched gene ontology (GO) associations are indicated on the *y* axis (left) while gene guides are listed on the right side. (**b**) Relative expression of the indicated viral genes in lung tissue from COVID-19 patient. Comparative analysis of differentially expressed mRNAs (**c**) and lncRNAs (**d**) in NHBE compared to patient-derived lung tissue.

Transcriptome data of lung tissue from patients with COVID-19 compared to healthy subjects from the Blanco-Melo study [32] were subjected to differential and biomarker discovery analysis (Figure 5a). Differentially expressed mRNAs and lncRNAs in COVID-19 compared to healthy control are listed in Supplementary Table S8. Functional enrichment studies show differentially expressed genes that positively correlate with an idealized cluster-specific expression profile in COVID-19 lung tissue, while enriched gene ontology (GO) associated with enriched GO terms are indicated on the *Y* axis. Color scales display marker gene correlation and differential gene expression (log2). Interestingly, several enriched GO classifications in the COVID-19 group were associated with immune response, negative regulation of viral genome replication, activation of JUN, and regulation of NFKB pathways (Figure 5a). Mapping and aligning RNA-seq data to the NCBI viral genome reference sequence revealed expression of orf1ab_8, S_14, M_51, ORF67a, ORF8_8 and N_65 viral genes (Figure 5b). There was no detected expression of ORF3a_9, E_10, ORF6_14, ORF7b and ORF10_9 viral genes in COVID-19 lung tissue (Figure 5b). In order to identify common genes and lncRNAs in the context of SARS-COV-2 infection, we compared the transcriptome of SARS-CoV-2 NHBE cells and lung tissue from patients with COVID-19, and identified 27 common upregulated and 867 common downregulated genes (Figure 5c). Similarly, comparing the lncRNA transcriptome also revealed 5 common upregulated and 57 common downregulated lncRNAs in SARS-CoV-2 NHBE and COVID-19 patient-derived lung tissue (Figure 5d), suggesting a plausible role for these genes and lncRNAs in host response to SARS-CoV-2 infection. The list of common altered mRNAs and lncRNAs in NHBE SARS-CoV-2 and COVID-19 is provided in Supplementary Table S9.

### 3.6. Upstream Regulator Predicts Upregulation of JAK-STAT and Interferon Cascade and miR-122 Functional Categories in Lung Tissue from COVID-19 Patient

**Figure 6:**
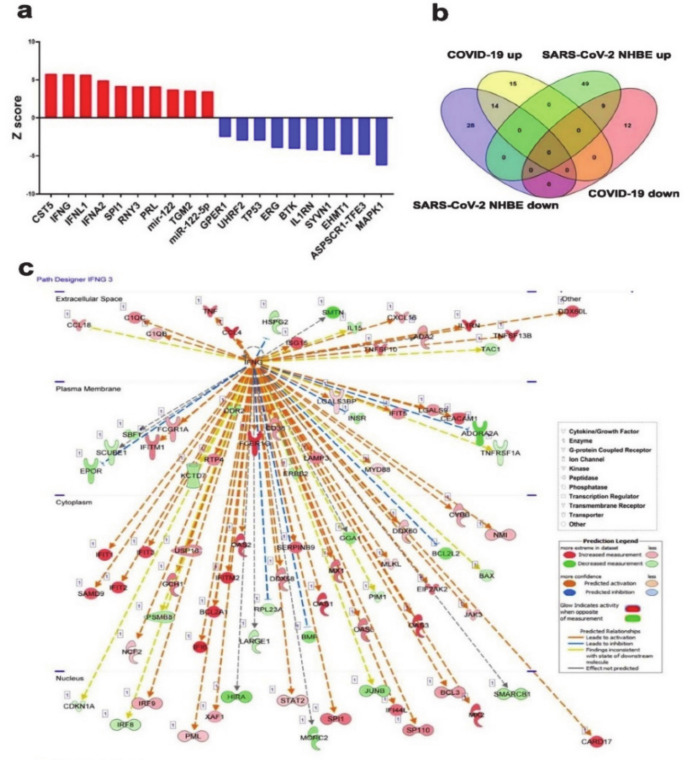
Mechanistic Network Analysis Predicts central role for IFNG in the host response of patients with COVID-19 infection. (**a**) Top ten activated and top ten inhibited upstream regulator networks in lung tissue derived from COVID-19 patients based on transcriptome and IPA analyses. (**b**) Venn diagram illustrating the overlap between activated and suppressed upstream regulator networks in SARS-COV-2 NHBE and lung tissue from COVID-19 patients based on RNA-seq and IPA analysis. (**c**) Illustration of the IFNG mechanistic network according to subcellular localization. Activation state is depicted according to color scale.

The top ten activated upstream regulator networks (CST5, IFNG, IFNL1, IFNA2, SPI1, RNY3, PRL, TGM2, miR-122 and miR-122-5p) in lung tissue derived from COVID-19 patient based on transcriptome and IPA analyses, revealed the enrichment of functions related to immune system associated JAK-STAT cascade, type 1 interferon receptor binding, cytokine receptor binding, and MHC 1 biosynthesis (Figure 6a and Supplementary Table S10). Likewise, the ten most inhibited upstream regulator networks were MAPK1, ASPSCR1-TFE3, EHMT1, SYVN1, IL1RN, BTK, ERG, TP53, UHRF2, and GPER1, respectively (Figure 6a). When comparing affected upstream regulator networks in SARS-CoV-2 NHBE and lung tissue from COVID-19 patients, we observed 14 common activated (IFNL1, CST5, SPI1, TRAP1, IFNG, NUPR1, TGM2, SMARCB1, RNY3, STAT1, IRF9, PRL, IFNA2, and Interferon α) and 9 common suppressed networks (TAP1, GPER1, TFEB, UHRF2, ASPSCR1-TFE3, IL1RN, BTK, SYVN1, and MAPK1, Figure 6b), suggesting changes observed in patient tissue are indeed inflicted by SARS-CoV-2 infection. IFNG mechanistic network illustrates gene expression and their interaction state as well as their subcellular localizing based on IPA. The network flow explains IFNG’s role and function in the extracellular space, plasma membrane, cytoplasm and nucleus. Arrows indicate direct interactions (activation or inhibition) between upstream and downstream pathway molecules. Molecule type is illustrated as per the indicated shapes. Activation state is depicted according to the color scale (Figure 6c).

### 3.7. Suppression of Viral Infection and Replication Based on Transcriptome and IPA Analysis of Lung-Derived Tissue from COVID-19 Patient

**Figure 7:**
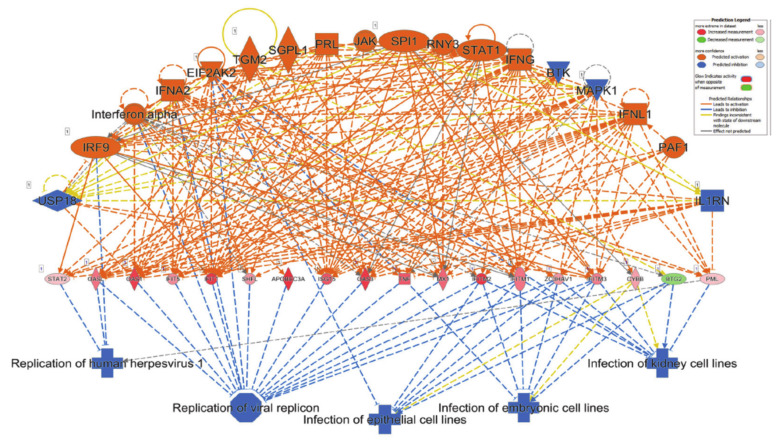
Suppression of viral infection and replication based on transcriptome and IPA analysis of lung-derived tissue from COVID-19 patient. Regulator effects network analysis based on IPA revealed suppression of viral infection and replication in lung tissue from patients with SASRS-COV-2 infection. Network highlights a role for BTK, EIF2AK2, IFNA2, IFNG, IFNL1, IL1RN, Interferon α, IRF9, JAK, MAPK1, PAF1, PRL, RNY3, SGPL1, SPI1, STAT1, TGM2 and USP18 in mediating these inhibitory effects as illustrated. Activation state is depicted according to the color scale.

The topmost enriched functional category based on RNA-seq from lung-derived tissue from COVID-19 patient using the regulator effect network analysis was the suppression of viral infection and replication (Figure 7).

**Figure 8:**
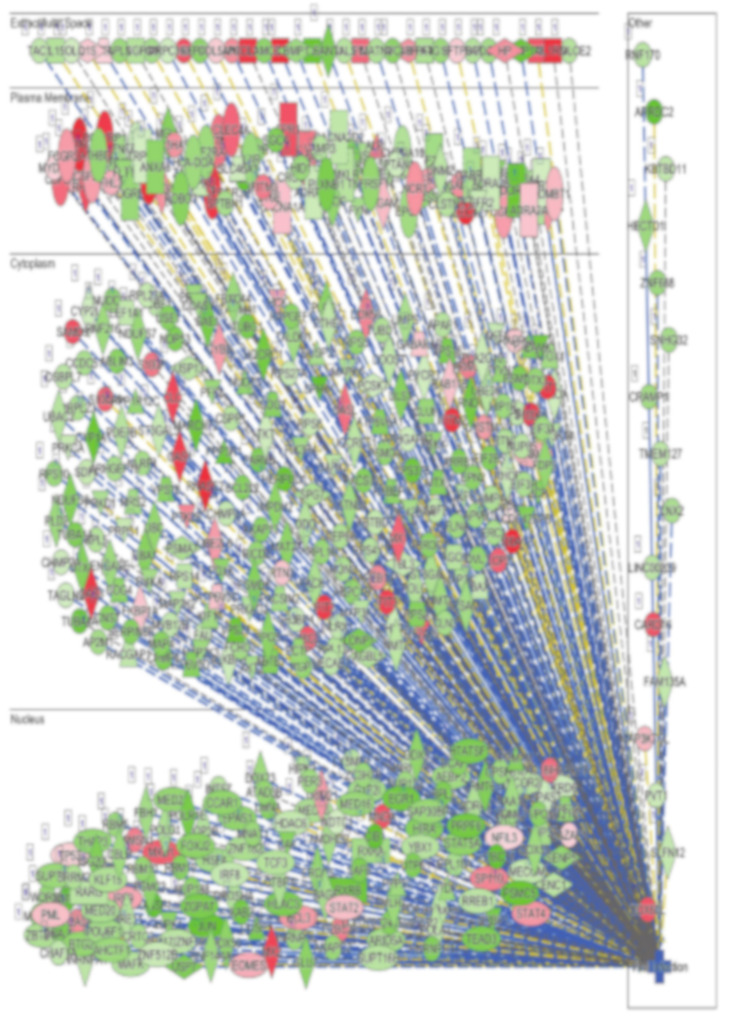
Illustration of the viral infection functional category according to subcellular localization based on transcriptome and IPA analysis of lung-derived tissue from COVID-19 patients.

The network combined differentially expressed potential upstream regulators (18, including 14 activated (orange; EIF2AK2, IFNA2, IFNG, IFNL1, Interferon α, IRF9, JAK, PAF1, PRL, RNY3, SGPL1, SPI1, STAT1 and TGM2), four inhibited (blue; BTK, MAPK1, IL1R1 and USP10)) and 18 mediator genes (including 17 increased and 1 decreased) in the middle of the hierarchy, which are involved in the inhibition of 5 major downstream effector functions such as viral infection and replication, and infection of epithelial cells. Among the 18 upstream regulators, IRF9, interferon α, IFNA2 and EIF2AK2 suppress downstream functions directly, as well as through the mediator genes, which collectively implies a massive IFNG response and subsequent predicted suppression of viral replication (Figure 7). Concordantly, disease and function analysis revealed viral infection as the most affected functional category in lung tissue from COVID-19 patients (Figure 8 and Supplementary Table S11). Cumulative complex network depicts the viral infection functional category according to subcellular localization. The majority of the green color associations indicate the down regulation and the minority in red indicate the upregulation of appropriate molecules in the extracellular space, plasma membrane, cytoplasm and nucleus (Figure 8).

## 4. Discussion

Since the recent outbreak of COVID-19, the number of affected individuals is rising expeditiously, claiming hundreds of thousands of lives thus far. Such a crisis calls for prompt responses from research communities to better understand and contain this rapidly spreading pandemic. In our current study, we explored recent in vitro and patient-derived transcriptome datasets to dissect the cellular and molecular changes in response to SARS-CoV-2 infection using state-of-the art bioinformatics pipelines. In vitro and patient-derived data revealed a central role for IFN signaling in the host response to SARS-CoV-2 infection. As part of our innate anti-viral defense, host cells release interferons as the first warning to a suspected infection. Interferons are a large group of signaling proteins (cytokines) that facilitate interference with viral replication as a defense mechanism, classed based on receptor signaling receptors and subsequent pathways, with some overlap. Our data shows a number of differentially expressed genes within the interferon type I and type III pathways in the SARS-CoV-2 infected cells compared to control NHBE cells. These genes (Supplementary Table S2) include STAT2 and IRF9, translocate to the nucleus and stimulate additional transcription of genes related to anti-viral responses, common to interferon type I and III [39,40]. In addition, other aberrantly expressed genes in response to viral infection include Vav1, which is member of interferon type I and III pathway, leading to cAMP Response Element-Binding Protein (CREB) mediated chromatin remodeling, also contributing to gene specific repression [41]. Furthermore, our data identified IRS2, mTOR genes, 4EBP1, EIF4A3, and RPS6, (components of type I and II signaling pathways) to be differentially expressed, affecting mRNA translation in response to interferon receptor signaling [42]. Blanco-Melo et al., also found a high level of chemokines induction, in concordance with our data, as well as high levels of type I and type III interferon response in human adenocarcinoma alveolar basal epithelial (A549) cells expressing ACE2, and in Calu-3 host cells infected with SARS-CoV-2 [32]. In these models, a significant interferon I and III response signature is seen, which is concordant with our findings.

Looking into changes in gene expression in lung biopsies from COVID-19 patients revealed substantial induction of immune response (*p* = 5.7 × 10^−35^), including innate immune response (*p* = 5.3 × 10^−29^). In particular, we observed the activation of antiviral defense (*p* = 4.7 × 10^−19^) mechanisms through upregulation of genes that inhibit viral replication (OAS1, OAS2, OASL, ISG15, MX1, APOBEC3A, C19orf66, EIF2AK2, IFIT1, IFITM1, IFITM2, IFITM3, LTF, TNF, and ZC3HAV1). Several of the upregulated genes are also known to play a role in preventing viral entry into host cells (FCN1, IFITM1, IFITM2, and IFITM3). A number of receptors which facilitate viral entry into host cells have been described. Interestingly in a patient with active viral gene transcription, we observed upregulation of several surface receptors implicated in viral entry into host cells (CD4, CD86, CD80, ACE2, CLDN1, CD55, CR1, and CLEC5A). Concordant with our findings, recent data revealed SARS-CoV-2 cell entry to depend on ACE2 and TMPRSS2 [7,24]. While ACE2 was upregulated in COVID-19 lung tissue, TMPRSS2 was severely downregulated (−7.1 FC, *p* = 0.007) suggesting downregulation of TMPRSS2 as a plausible mechanism through which the host counteracts SARS-CoV-2 infection. It is not clear why ACE2 expression was unregulated in lung tissue from COVID-19 patient. It is possible that ACE2 expression is induced in response to interferon signaling. In fact, analyzing data from NHBE cells treated with INFB revealed a time-dependent increase in ACE2 expression in response to INFB treatment (Supplementary Figure S2).

Looking into commonalities in upstream analysis between the NHBE and COVID-19 data, our data revealed 14 common activated (IFNL1, CST5, SPI1, TRAP1, IFNG, NUPR1, TGM2, SMARCB1, RNY3, STAT1, IRF9, PRL, IFNA2, and Interferon α) and 9 common suppressed (TAP1, GPER1, TFEB, UHRF2, ASPSCR1-TFE3, IL1RN, BTK, SYVN1, and MAPK1) networks. Interestingly, our data highlighted a role for the activation states of PAF1, IFNL1, IFNG, STAT1, RNY3, SPI1, JAK, PRL, SGPL1, TGM2, EIF2AK2, IFNA2, interferon α, and IRF9 as the upstream regulators leading to subsequent activation of CYBB, IFITM3, ZC3HAV1, IFITM1, IFITM2, MX1, TNF, OAS3, ISG15, APOBEC3A, IFIT1, OAS1, OASL, and STAT2 activation, collectively leading to inhibition of viral genome replication. Our data also highlights a central role for CST5 and IFNG activation and suppression of MAPK1 and Interleukin-1 receptor antagonist (IL1RN) in the host response against SARS-COV-2 infection.

Downregulation of the Mitogen-activated protein kinase 1 (MAPK1) network was observed in SARS-CoV-2 infected NHBE as well as in COVID-19 lung tissue. MicroRNA translation of IFN-stimulated genes is additionally regulated through the Mitogen-activated protein kinase (MAPK) pathway [43], as well as a variety of essential cell processes including mitosis, cell survival, apoptosis, metabolism and cell differentiation [44]. MAPK1, or ERK2, was found to be suppressed in the in vitro model and patient data in our current study. Upon phosphorylation, MAPK1 subsequently phosphorylates a number of transcription factors including FOS, MYC, EGR-1, Elk-1, and JUN [45]. Therefore, its suppression will ultimately affect transcriptional activation of further downstream genes. Research into the Ebola virus by Zampieri et al., show glycoprotein mediated cytotoxicity as a result of MAPK signaling cascade. This inhibition of ERK2 was found to negatively affect cellular viability and integrin expression [46]. This is also in concordance with other studies that show inhibition of the ERK pathway as a result of hepatitis C virus (HCV) and HIV type 1 infection [47,48].

In addition to protein coding genes, our data also highlighted a number of lncRNAs which were differentially expressed in COVID-19 patient-derived lung tissue. For instance, using marker finder algorithm, upregulation of AC131011.2, AC007298.2, AC002398.2, AC022966.2, AC006064.4, AC099343.4, AC007032.1, AL034397.3, AC008537.4 and downregulation of LINC01089, LINC00115, AC027288.3, AC103706.1, AC022098.1, AC020915.3, AC007192.2, AP002840.2, AC018690.1, AC015819.1, AC009318.2, AC245140.2, AC097382.3, and AL035587.2 was associated with COVID-19 infection. Interestingly, we observed upregulation of metastasis-associated lung adenocarcinoma transcript 1 (MALAT1) and Nuclear-enriched autosomal transcript 1 (NEAT1) lncRNAs in NHBE cells in response to SARS-CoV-2 infection. Despite the emergence of lncRNAs as critical mediators of various biological processes, mechanistic roles for many lncRNAs in viral infection are still poorly understood. Studies on MALAT1 have shown this lncRNA to be overexpressed in several cancer tissues, associated with high rates of metastasis, and poor prognosis in lung cancer [49], breast cancer [50], colon cancer [51] and esophageal cancer [52], as well as several other cancer types. MALAT1 has also been implicated in the regulation of histone acetylation [53], endothelial to mesenchymal transition (EMT) [54], and cardiac inflammation and dysfunction [55]. Emerging research has highlighted yet another role for MALAT1 lncRNA in viral infection and innate immune processes, affirming the crucial roles it plays in many biological processes. Wei et al., investigates the role of MALAT1 in inflammatory injury following lung transplant, which could give plausible indicators for the role of MALAT1 in inflammation injury following SARS-Cov-2 infection. Interestingly, silencing MALAT1 alleviated inflammatory injury by inhibiting neutrophil chemotaxis and immune cell infiltration to the site of infection [56]. They suggest this could regulate the progression of acute lung injury through NF-kB and p38 MAPK pathways [57], however is it also plausible that the inhibition of neutrophil chemotaxis lightens the burden of cytokine storms in lung inflammation injury. Bhattacharyya et al., describe the role of MALAT1 in two flaviviruses; Japanese encephalitis virus (JEV) and West Nile virus (WNV). Neuro2a cells treated with these viruses show MALAT1 overexpression, promoting inflammatory response [58].

MALAT1, along with lncRNA NEAT1, have been shown to be potential biomarkers for HIV infection, after the detection of high levels of both lncRNAs in peripheral blood mononuclear cells (PBMCs) upon infection [59]. Qu et al., found MALAT1 to promote HIV-1 transcription and infection by alleviating the epigenetic silencing of HIV-1 transcription via its interaction with enhancer of zeste homolog 2 (EZH2), which binds the HIV-1 promoter [60]. *Neat1* knockout mice present enhanced inflammation through the activation of NLRP3 and NLRC4 inflammasomes, promoting inflammatory mediated cell death in vivo [61]. In addition to this, NEAT1 has also been shown to be involved in HIV-1 replication in infected cells. The knockdown of NEAT1 enhanced viral production by promoting nucleus-to cytoplasm export of HIV-1 mRNA transcripts in HeLa cells [62]. The current data provides us with plausible indicators surrounding the involvement of such lncRNAs in the progression of SARS-Cov-2 infection. The specific mechanisms, whether it be via the regulation of inflammation, epigenetic silencing of target genes or dysregulation of gene expression are yet to be deciphered. Further research into the roles of MALAT1 and NEAT1 in the context of COVID-19 will be valuable in further understanding the mechanism behind disease progression in the perusal of potential biomarkers and therapeutic intervention.

## 5. Conclusions

Our results highlighted activation of IFN and inflammatory response as the main hallmark associated with SARS-CoV-2 infection, and identified several differentially expressed lncRNAs during the course of infection, which could serve as disease biomarkers, while their precise role in the host response to SARS-CoV-2 remains to be investigated. Interferon and inflammatory response to SARS-CoV-2 infection might provide explanation to cytokine storms associated with severe COVID-19 cases.

## Supplementary Materials

The following are available online at https://www.mdpi.com/2073-4425/11/7/760/s1. Figure S1: Expression of selected antiviral host response genes in CAlu-3 cells infected with SARS-CoV-2. Figure S2: Expression of ACE2 in NHBE cells response to IFNB based on RNASEQ analysis. Table S1. Mapped reads expression values (TPM) for mRNA, lncRNA, and viral genes in all samples analyzed in current study. Table S2: Differentially expressed genes in SARS-CoV-2 infected vs. control NHBE cells. Table S3: Clustering marker gene correlations for SARS-CoV-2 infected and control NHBE cells. Table S4: Affected canonical pathways based on IPA analysis of differentially expressed mRNAs in SARS-CoV-2 infected NHBE cells. Table S5: Upstream regulator analysis based on IPA analysis of differentially expressed mRNAs in SARS-CoV-2 infected NHBE cells. Table S6: Disease and function analysis based on IPA analysis of differentially expressed mRNAs in SARS-CoV-2 infected NHBE cells. Table S7: Differentially expressed lncRNA genes in NHBE SARS-CoV-2 infected cells. Table S8: Differentially expressed genes in lung tissue from COVID-19 compared to healthy subject. Table S9. Common upregulated and downregulated lncRNAs in SARS-CoV-2 NHBE and COVID-19 patient. Table S10: Affected upstream regulator networks in lung tissue from COVID-19 patient compared to healthy subject based on RNA-seq and IPA. Table S11: Disease and function analysis based on IPA analysis of differentially expressed genes in lung tissue from COVID-19 patient.
