# Supplementary material for: Protein Coding and Long Noncoding RNA (lncRNA) Transcriptional Landscape in SARS-CoV-2 Infected Bronchial Epithelial Cells Highlight a Role for Interferon and Inflammatory Response
Source: Genes (Basel). 2020 Jul 7;11(7):760. doi: 10.3390/genes11070760 (PMC7397219; doi:10.3390/genes11070760)
Supplement: Supplementary file 1 [file genes-11-00760-s001.zip › Supplementary Figure 1.pdf]

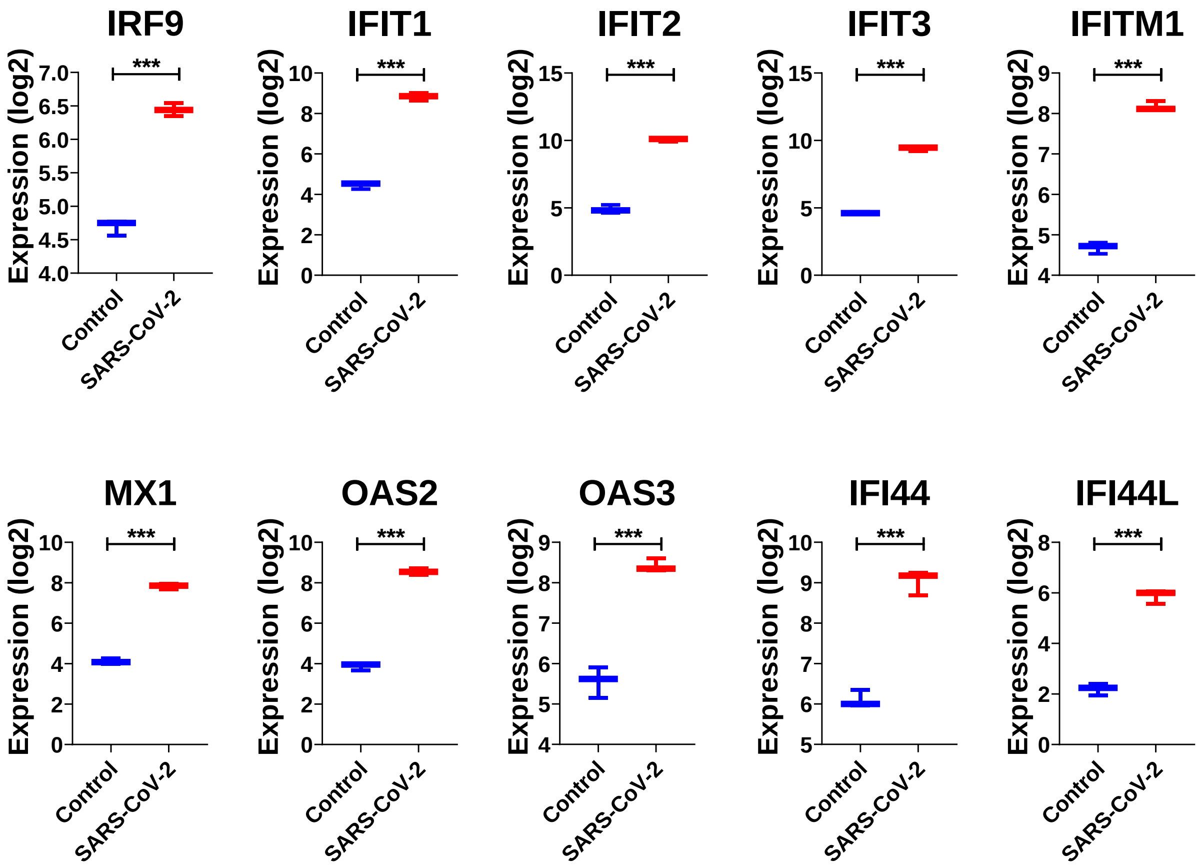

Supplementary figure 1. Expression of selected antiviral host response genes in CAlu-3 cells infected with SARS-CoV-2. Expression data from Calu-3 cells (SRX8089279, SRX8089280, and SRX8089281) compared to control calu-3 cells (SRX8089276, SRX8089277, and SRX8089278). Data were aligned to hg38 in CLC genomics workbench 20.0. Data are presented as scattered plot with the mean and SD indicated (n=3). ***P< 0.001
