# Supplementary material for: Protein Coding and Long Noncoding RNA (lncRNA) Transcriptional Landscape in SARS-CoV-2 Infected Bronchial Epithelial Cells Highlight a Role for Interferon and Inflammatory Response
Source: Genes (Basel). 2020 Jul 7;11(7):760. doi: 10.3390/genes11070760 (PMC7397219; doi:10.3390/genes11070760)
Supplement: Supplementary file 1 [file genes-11-00760-s001.zip › Supplementary figure 2.pdf]

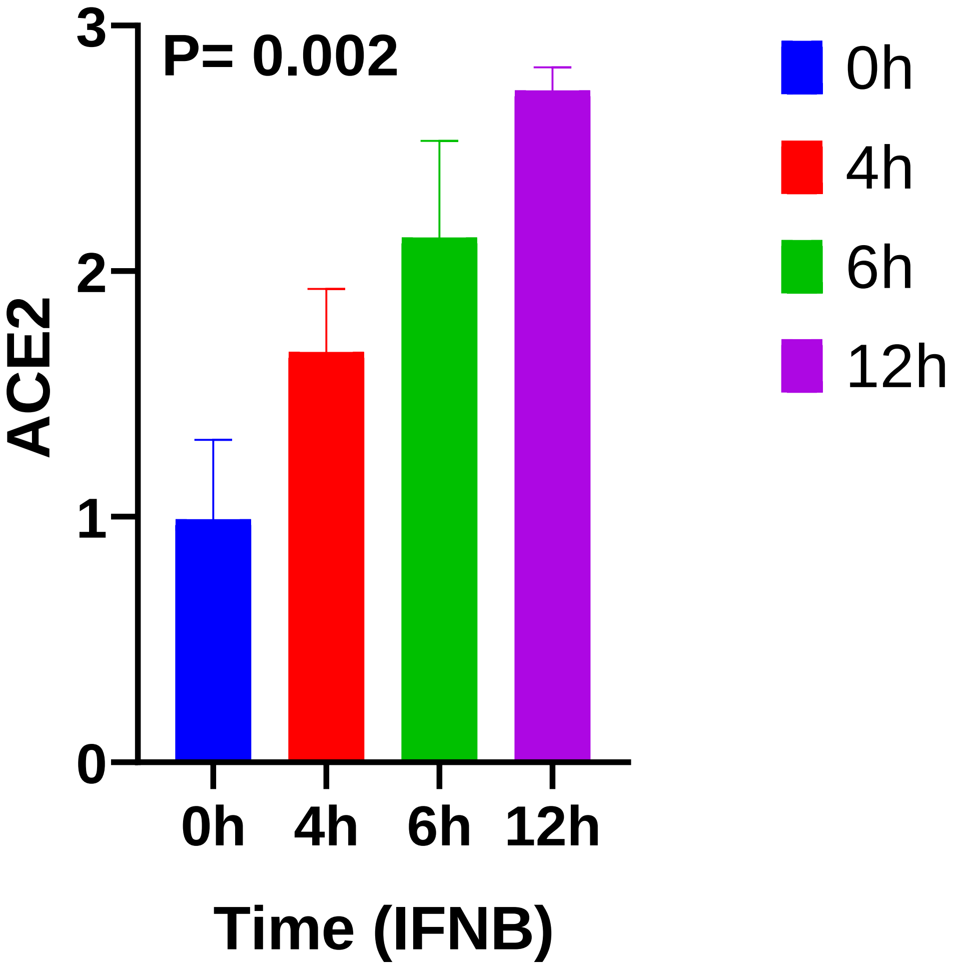

Supplementary figure 2. Expression of ACE2 in NHBE cells response to IFNB based on RNASEQ analysis.
